# Efficacy of recombinant H5 vaccines delivered *in ovo* or day of age in commercial broilers against the 2015 U.S. H5N2 clade 2.3.4.4c highly pathogenic avian Influenza virus

**DOI:** 10.1186/s12985-023-02254-1

**Published:** 2023-12-15

**Authors:** Darrell R. Kapczynski, Klaudia Chrzastek, Revathi Shanmugasundaram, Aniko Zsak, Karen Segovia, Holly Sellers, David L. Suarez

**Affiliations:** 1 Exotic and Emerging Avian Viral Diseases Research Unit, U.S. National Poultry Research Center, Agricultural Research Service, U.S. Department of Agriculture, 934 College Station Road, 30605 Athens, GA U.S.; 2 Department of Population Health, College of Veterinary Medicine, The University of Georgia, 956 College Station Road, 30602 Athens, Athens, GA U.S.

**Keywords:** Chicken, Broiler, Highly pathogenic avian Influenza virus, Vaccine efficacy, Viral shedding, Recombinant vaccine

## Abstract

**Background:**

Avian influenza is a highly contagious, agriculturally relevant disease that can severely affect the poultry industry and food supply. Eurasian-origin H5Nx highly pathogenic avian influenza viruses (HPAIV) (clade 2.3.4.4) have been circulating globally in wild birds with spill over into commercial poultry operations. The negative impact to commercial poultry renewed interest in the development of vaccines against these viruses to control outbreaks in the U.S.

**Methods:**

The efficacy of three recombinant H5 vaccines delivered *in ovo* or day of age were evaluated in commercial broilers challenged with the 2015 U.S. H5N2 clade 2.3.4.4c HPAIV. The recombinant vaccines included an alphavirus RNA particle vaccine (RP-H5), an inactivated reverse genetics-derived (RG-H5) and recombinant HVT vaccine (rHVT-AI) expressing H5 hemagglutinin (HA) genes. In the first experiment, *in ovo* vaccination with RP-H5 or rHVT-AI was tested against HPAI challenge at 3 or 6 weeks of age. In a second experiment, broilers were vaccinated at 1 day of age with a dose of either 10^7^ or 10^8^ RP-H5, or RG-H5 (512 HA units (HAU) per dose).

**Results:**

In experiment one, the RP-H5 provided no protection following *in ovo* application, and shedding titers were similar to sham vaccinated birds. However, when the RP-H5 was delivered *in ovo* with a boost at 3 weeks, 95% protection was demonstrated at 6 weeks of age. The rHVT-AI vaccine demonstrated 95 and 100% protection at 3 and 6 weeks of age, respectively, of challenged broilers with reduced virus shedding compared to sham vaccinated birds. Finally, when the RP-H5 and rHVT vaccines were co-administered at one day of age, 95% protection was demonstrated with challenge at either 3 or 6 weeks age. In the second experiment, the highest protection (92%) was observed in the 10^8^ RP-H5 vaccinated group. Significant reductions (p < 0.05) in virus shedding were observed in groups of vaccinated birds that were protected from challenge. The RG-H5 provided 62% protection from challenge. In all groups of surviving birds, antibody titers increased following challenge.

**Conclusions:**

Overall, these results demonstrated several strategies that could be considered to protected broiler chickens during a H5 HPAI challenge.

## Background

During the past 40 years, the number and frequency of highly pathogenic avian influenza (HPAI) outbreaks in poultry worldwide has steadily increased [1, 2]. In North America, a HPAI clade 2.3.4.4c H5N_x_ HPAI epizootic began in November 2014 when Eurasian HPAI H5N8 and H5N2 was detected in wild and backyard birds [3–5]. The following year, infections with the H5N2 HPAI virus devastated poultry production in the Midwest of USA, primarily affecting egg-laying chickens and turkeys, with approximately 47 million poultry dead or euthanized [6]. A stamping-out policy was employed to control the epizootic, without vaccination, which was declared over in June 2015. In January of 2022, isolation of H5N1 clade 2.3.4.4b HPAI occurred in an American Widgeon in South Carolina (https://www.aphis.usda.gov/aphis/newsroom/stakeholder-info/sa_by_date/sa-2022/hpai-sc). This event began on the east coast, primarily in wild birds, and subsequently infected commercial and backyard poultry. Over 171 commercial and 108 backyard flocks were confirmed positive for H5Nx HPAI, affecting more than 37.5 million birds at that time. (https://www.aphis.usda.gov/aphis/ourfocus/animalhealth/animal-disease-information/avian/avian-influenza/hpai-2022).

During the H5Nx outbreak in 2015, multiple studies developed and tested commercial and experimental H5 vaccines against avian influenza virus (AIV) to demonstrate protective efficacy against the clade 2.3.4.4 H5Nx HPAI virus [7–11]. When used properly and in conjunction with other control measures, vaccines can be an effective method to control clinical disease and reduce virus transmission, and has led to successful HPAI eradication [12, 13]. More recently, effective application of vaccines for either H5 or H7 HPAI have significantly reduced some HPAI viruses in China, particularly H7N9 [14, 15]. One key aspect of this vaccine approach is the continued updating of seed strain utilized in the vaccine to match field virus HA as it continually evolves.

Previous HPAI epizootics have occurred in vaccinated chicken flocks [13]. This may be the result of improper administration, lack of timely seroconversion before HPAI exposure, antigenic mismatch or use of poor quality vaccine. Qualities of a proper poultry vaccine include efficacy, safety, onset of immunity, ease of application, decrease virus replication and transmission potential, cost, maternal antibody consideration, and the ability to distinguish infected versus vaccinated animals (DIVA strategy) [12, 13].

Our previous vaccine studies in chickens demonstrated that older USDA H5 vaccine bank strains (e.g. Tk/WI/68(H5N9)) provided limited protection against clade 2.3.4.4 H5N2 HPAI, signifying the need for new vaccines [7]. In addition, we have demonstrated variable protection with a replication-deficient alphavirus vaccine (RP-H5) and a recombinant turkey herpes virus vaccine (rHVT-AI) following clade 2.3.4.4 H5 challenge in laying hens and turkey [8, 9]. No vaccine studies have been performed with broiler chickens. Although major differences in response from layer chickens are not anticipated, the short production life of commercial broilers of 6–7 weeks requires a different strategy for vaccination to provide earlier protection. Specifically, the vaccination at day of age, if effective, allows for more efficient vaccination at the hatchery before birds are placed in the field and potentially offers earlier protection.

The objective of the current study was to investigate protection of broiler-type birds using recombinant AI vaccines delivered *in ovo* or at day-of-age followed by challenge at 3 or 6 weeks of age with a 2015 North American HPAI virus. The study demonstrates that a single dose of rHVT-AI or RP-H5 provided above 90% protection following lethal challenge. In addition, a dose response was observed with the RP-H5 vaccine when delivered at one-day of age. A combination of the two vaccines delivered at day of age also protected chickens from a H5N2 HPAIV challenge. The results provide a framework for consideration of HPAI vaccination of broilers in the U.S.

## Materials and methods

### Viruses

The highly pathogenic A/turkey/Minnesota/12,582/2015 (TK/MN/15) H5N2 clade 2.3.4.4c was used as the challenge virus. Virus was propagated in specific pathogen free (SPF) embryonated chicken eggs according to standard procedures [16]. Allantoic fluid containing virus was harvested for titration of HPAI challenge virus using standard procedures in eggs [8]. All experiments using HPAI viruses, including work with animals, were approved by the institutional biosecurity committee and the institutional animal care and use committee (IACUC), and were performed in animal biosecurity level-3 enhanced (ABSL3E) facilities at the U.S. National Poultry Research Center (USNPRC), Athens, Georgia.

### Vaccines

Three recombinant vaccines were utilized in these studies. A replication-deficient alphavirus RNA particle vaccine (RP-H5) was provided by the manufacturer (Merck Animal Health, Ames, IA). The H5 HA gene from A/Gyrfalcon/Washington/41088-6/2014 H5N8 clade 2.3.4.4c, with a modification of the HA cleavage site to a LPAI virus genotype, was constructed into the replication-deficient alphavirus particles. A rHVT vaccine encoding the H5 gene was provided by the manufacturer (CEVA Animal Health, Lenexa, KS). This vaccine was constructed by inserting the HA gene of A/swan/Hungary/4999/2006 H5N1 clade 2.2 strain into the genome of HVT FC-126 strain (rHVT-AI). The cleavage site of the HA gene used in the rHVT-AI was also altered to a typical LPAI virus strain. Finally, an RG-H5 virus was constructed using the HA gene from A/Gyrfalcon/Washington/41088-6/2014 H5N8 clade 2.3.4.4c which was *de novo* synthesized (Integrated DNA Technologies, Coralville, Iowa) with a modification of the HA cleavage site to be compatible with a H5 low pathogenic avian influenza (LPAI) virus [7]. Using the modified H5 gene and the remaining 7 gene segments from the Puerto Rico/8/1934 (PR8) egg adapted virus inserted in the plasmids, virus was rescued and passaged into specific pathogen free embryonated chicken eggs [8]. The RG-H5 virus were prepared to contain 512 HAU per dose (0.2 ml) mixed (70/30) in Montanide ISA VG70 oil emulsion adjuvant (SEPPIC, Inc., Fairfield, NJ) and delivered SQ in the neck [8].

### Experiment 1

Commercial broiler chickens (Fieldale Farms, Cordelia, GA) were used in all studies. All *in ovo* vaccinations were delivered on day 18 day of incubation to embryonation chicken eggs using an Embrex Inovoject® (Durham, NC) at the Poultry Diagnostic Research Center (PDRC), The University of Georgia, Athens, Georgia. All animal work was approved by the University of Georgia IACUC committee. Vaccine groups are shown in Table 1. Group 1 contained 30 birds that received sham *in ovo* vaccination with 0.1 ml of PBS. Birds in these groups were challenged at 3 (20 birds) and 6 (10 birds) weeks of age (woa) (Table 1). Group 2 contained 30 birds that received RP-H5 *in ovo* in 0.1 ml at a dose of 10^8^ virus particles per dose. Twenty birds were challenged at 3 woa and 10 birds challenged at 6 woa. Group 3 contained 30 birds that received rHVT-AI *in ovo* in 0.1 ml according to the manufacturers recommended dose. Twenty birds were challenged at 3 woa and 10 birds challenged at 6 woa. Group 4 contained 10 birds that received *in ovo* vaccination with 0.1 ml of RP-H5 (10^8^) particles and booster vaccination with 0.1 ml of RP-H5 (10^8^) particles given via subcutaneous injection into the neck at 3 woa. These birds were challenged at 6 woa. Group 5 contained 30 birds vaccinated SQ at 1 day of age with 0.2 ml of RP-H5 (10^8^ particles in 0.1 ml) and rHVT-AI (1 dose in 0.1 ml). Twenty birds in this group were challenged at 3 woa and 10 birds were challenged at 6 woa.

**Table 1:** Vaccine groups and schedules in challenge experiments #1 and #2

#1Group	Route	Dose	Vaccine	Challenge ^d^	N ^e^
G1	In ovo	0.1 ml	Sham-PBS	3 & 6 woa	20/10
G2	In ovo	0.1 ml	RP-H5^c^	3 & 6 woa	20/10
G3	In ovo	0.1 ml	rHVT-AI	3 & 6 woa	20/10
G4	In ovo / boost^a^	0.1 ml / SQ	RP-H5^c^ / RP-H5^c^	6 woa	10
G5	SQ^b^	0.2 ml / SQ	RP-H5^c^ / rHVT-AI	3 & 6 woa	20/10
#2Group	Route	Dose	Vaccine	Challenge	N
G1	SQ	0.2 ml	PBS	3 woa	11
G2	SQ	0.2 ml	RP-H5 (10^7^)	3 woa	13
G3	SQ	0.2 ml	RP-H5 (10^8^)	3 woa	13
G4	SQ	0.2 ml	RG-H5	3 woa	13

^a^ Boost at 3 weeks of age

^b^ SQ = subcutaneous (1 day of age)

^c^ 10^8^ particles per bird

^d^ woa = weeks of age

^e^ N = number of birds challenged at 3 woa/6 woa

### Experiment 2

Vaccine groups are shown in Table 1. Group 1 contained 11 birds that received sham vaccination with 0.2 ml of PBS at one-day of age. Group 2 contained 13 birds that received RP-H5 in 0.2 ml SQ into the neck at a dose of 10^7^ virus particles per dose at one-day of age. Group 3 contained 13 birds that received vaccination with 0.2 ml of 10^8^ RP-H5 particles given via SQ injection into the neck at one-day of age. Finally, group 4 contained 13 birds which were vaccinated at one-day of age with RG-H5 virus containing 512 HAU per dose (0.2 ml) mixed (70/30) in Montanide ISA VG70 oil emulsion adjuvant (SEPPIC, Inc., Fairfield, NJ) and delivered SQ in the neck [7].

### Challenge and evaluation of protection

Prior to challenge, birds in both experiments were moved into ABSL3E high biocontainment facilities (USNPRC) to allow acclimation. Birds were challenged intranasally (IN) at 3 woa with 0.1 ml containing 10^6.5^ 50% embryo infectious doses (EID_50_) of TK/MN/15 clade H5N2 clade 2.3.4.4c HPAI. After challenge, birds were observed daily, and a record of number of mortalities was kept. Serum was collected prior to and 10 days post-challenge (dpc), and oropharyngeal swabs were collected 2 and 4 dpc to quantitate virus shedding. Surviving birds were euthanized 14 dpc. All birds received water and feed *ad libitum* at UGA, PDRC and USDA, USNPRC. All animal work at the USNPRC was approved by IACUC committee.

### Determination of virus shedding

Oropharyngeal and cloacal swabs were collected in 1 ml of sterile brain heart infusion medium and kept frozen at -70 °C. Viral RNA was extracted using the MagMAX AI/ND Viral RNA Isolation Kit (Ambion, Austin, TX). Quantitative real-time RT-PCR (qRRT-PCR) was performed, as previously described [17]. Briefly, qRRT-PCR targeting the influenza M gene was conducted using AgPath-ID one-step RT-PCR Kit (Ambion) on the ABI 7500 Fast Real-Time PCR system (Applied Biosystems, Carlsbad, CA). For quantification of viral shedding, a standard curve was established with viral RNA extracted from the titrated challenge virus TK/MN/15. Results were reported as mean log_10_ EID_50_/ml ± SEM equivalents and the lower limit of detection being 10^0.9^ EID_50_/ml.

### Determination of serum hemagglutination inhibition (HI) activity

Blood was collected from birds via brachial vein venipuncture on 0 and 14 dpc in experiment 1, and 0 and 10 dpc in experiment 2. Serum was isolated and stored at -20 °C before use. Serum HI activity was determined using BPL inactivated TK/MN/15 antigen (clade 2.3.4.4c), as described previously [18]. HI titers are reported as log_2_ values, with 3 log_2_ being the minimum titer considered as positive.

### Statistical analysis

Kaplan–Meier survival curves were generated with Prism 8.0 (GraphPad Co., San Diego, CA). The Mantel Cox Log-rank test was used to compare survival curves between virus challenged groups (Prism). Statistical differences in mean and standard error of the mean between virus and HI titers were analyzed using ANOVA (Prism). Chicks with HI titers of 0 were assigned a value of 1 for statistical purposes. Lower case letters indicate statistical significance between compared groups. All statistical tests used *P* < 0.05 for significance.

## Results

### Survival of broilers following lethal clade 2.3.4.4 H5N2 HPAIV challenge in experiment 1 and 2

The RP-H5, RG-H5 and rHVT-AI vaccines tested encode HA genes that were 99%, 99% and 90% similar, respectively, to the challenge virus HA (data not shown) [7]. In all challenge trials, broilers were intranasally challenged with 10^6.5^ EID_50_ of TK/MN/15 H5N2 HPAI and monitored daily for morbidity and mortality.

**Fig. 1:**
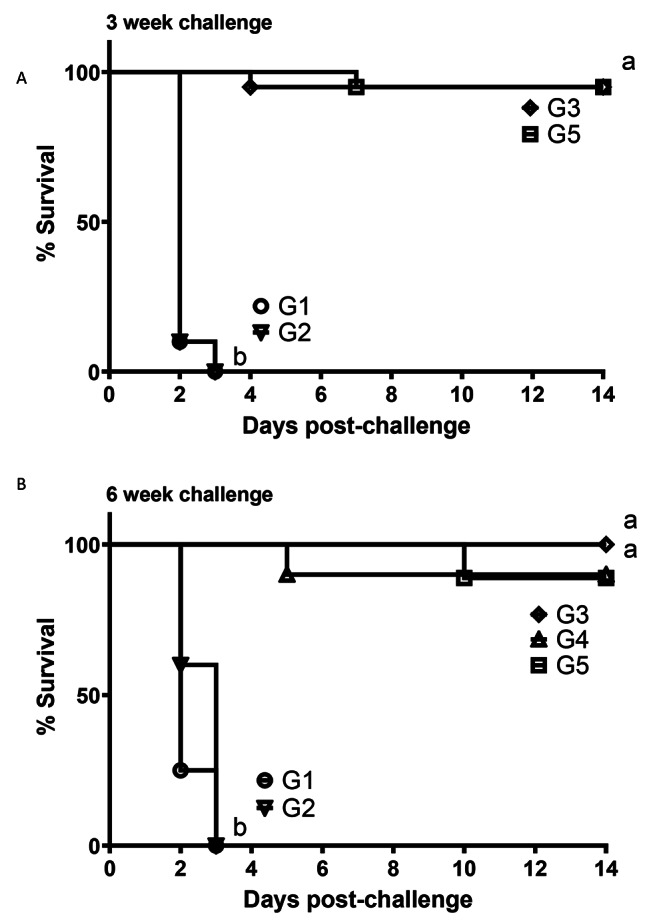
Kaplan-Meier survival plots of broiler chicks immunized with recombinant vaccines and challenged with HPAI virus in experiment 1. Eighteen day-old embryonated eggs were vaccinated *in ovo* with PBS (G1), RP-H5 (G2), rHVT-AI (G3), RP-H5 *in ovo* and boost at 3 weeks of age (G4), or combined rHVT-AI and RP-H5 delivered subcutaneously SQ at one day-of-age (G5). Birds were challenged with A/turkey/Minnesota/12,582/2015 H5N2 clade 2.3.4.4c HPAI virus at either 3 weeks of age (Fig. 1**A**) or 6 weeks of age (Fig. 1**B**). Statistical differences in percent survival were determined using the Mantel-Cox log-rank test and indicated by different lowercase letters (*P* < 0.05)

In experiment 1, vaccination significantly (*P* < 0.05) protected broilers against HPAI challenge, at both challenges at 3 and 6 woa, although differences were observed in the level of protection depending on the vaccine used (Fig. 1). *In ovo* vaccination with rHVT-AI (G3) or day of age with combined vaccines (G5) resulted in 95% protection in birds challenged at 3 woa (Fig. 1A). All sham (G1) and RP-H5 (G2) *in ovo* vaccinated birds were dead by 3 dpc. Some of the sham and RP-H5 *in ovo* vaccinated broilers displayed clinical sign of disease including depression, lethargy, diarrhea, and torticollis when challenged at 3 woa.

Similar results were observed when broilers were challenged at 6 woa. In that experiment, the rHVT-AI (G3) *in ovo* vaccinated birds were 100% protected from lethal H5N2 AIV challenge (Fig. 1B). Similarly, the RP-H5/RP-H5 (G4) *in ovo*/boosted broilers were 95% protected from challenge. Broilers that received RP-H5/rHVT-AI (G5) at 1 day-of-age were also 95% protected. As in the previous trail, the *in ovo* sham (G1) and RP-H5 (G2) vaccinated birds were all dead by 3 dpc. Clinical signs observed in these groups were as above.

**Fig. 2:**
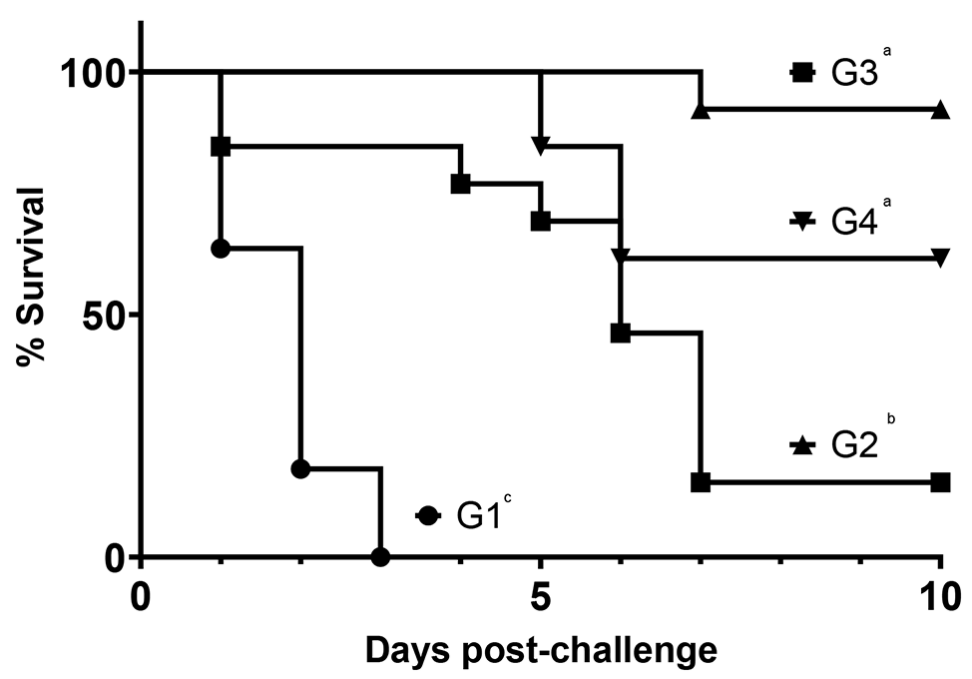
Kaplan-Meier survival plots of one-day of age broiler chicks immunized with recombinant vaccines and challenged with HPAI virus in experiment 2. One-day old commercial chicks were vaccinated SQ with PBS (G1), RP-H5 (10^7^) (G2), RP-H5 (10^8^) (G3), or RG-H5 (G4). Birds were challenged with A/turkey/Minnesota/12,582/2015 H5N2 clade 2.3.4.4c HPAI virus at 3 weeks of age. Statistical differences in percent survival were determined using the Mantel-Cox log-rank test and indicated by different lowercase letters (*P* < 0.05)

In experiment 2, significant differences in survival were observed between the different vaccine groups (Fig. 2). All sham-vaccinated birds (G1) were dead by 3 dpc. Birds receiving 10^7^ RP-H5 particles subcutaneously (G2) were protected at 15%, with most birds dying between day 4 and 7 pc. Groups of birds vaccinated with 10^8^ RP-H5 particles (G3) were protected at 92%, with only one bird dying at 7 dpc. In G4, birds receiving the RG-H5 vaccine were protected at 62% from lethal challenge.

### Virus shedding after challenge with H5N2 HPAIV in experiment 1 and 2

**Fig. 3:**
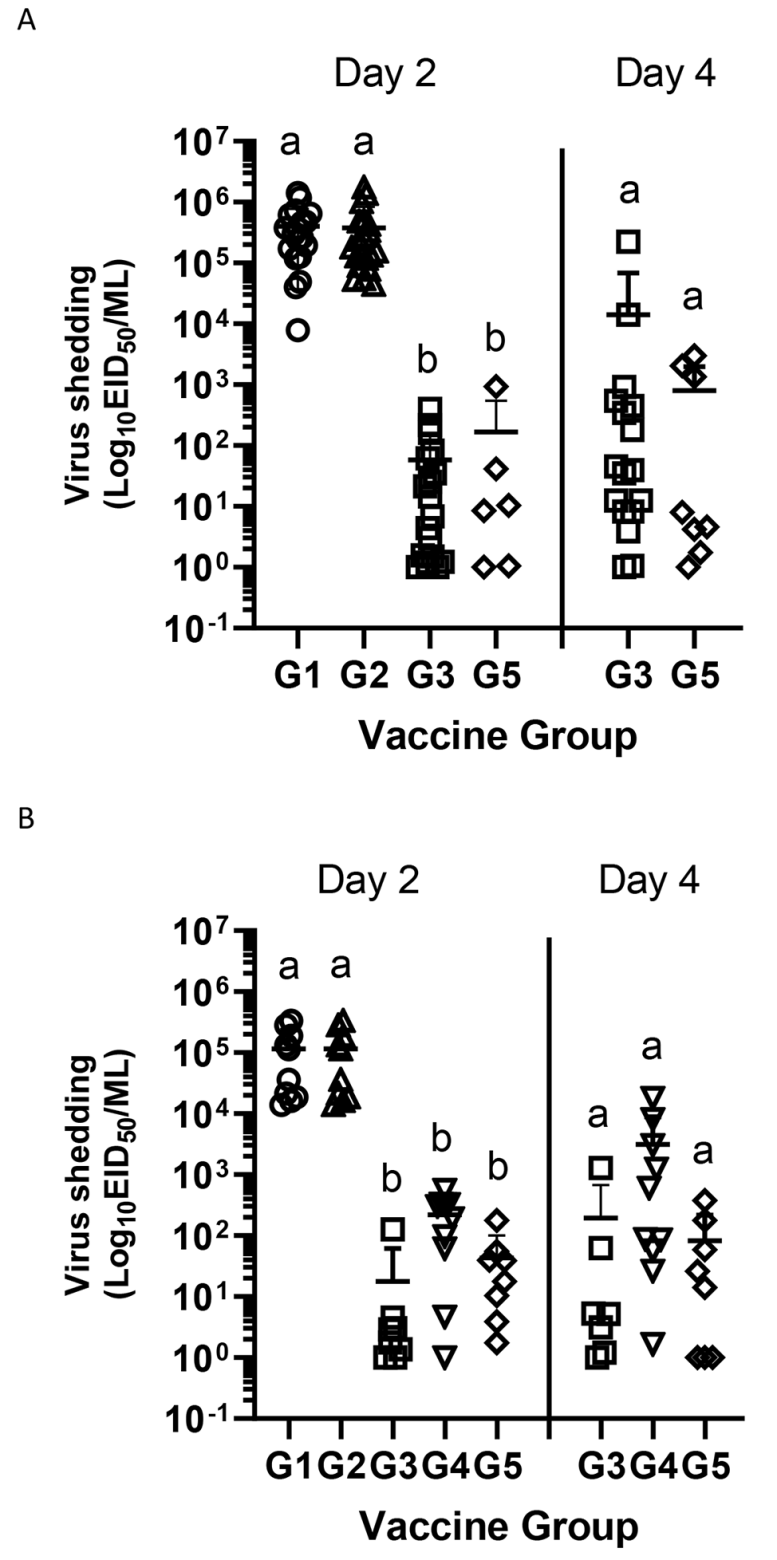
Viral titers from oral swabs in broiler chicks immunized with recombinant vaccine and challenged with HPAI virus in experiment 1. G1, G2, G3: 18-day-old embryonated eggs were vaccinated *in ovo* with PBS (Sham control; G1) or RP-H5 (G2) or rHVT-AI (G3). G4: Birds in G2 group were given an additional RP-H5 booster dose subcutaneously at 3 weeks of age. G5: Day old birds were vaccinated with both rHVT-AI and RP-H5 vaccine. Birds were challenged with A/turkey/Minnesota/12,582/2015(H5N2) clade 2.3.4.4 HPAI virus at either 3 weeks of age (Fig. 3**A**) or 6 weeks of age (Fig. 3**B**). Swabs were analyzed at 2 and 4 dpc using qRRT-PCR. Viral titers are expressed as log_10_ EID_50_/ml. Statistical differences are indicated by different lowercase lettering (*P* < 0.05)

**Fig. 4:**
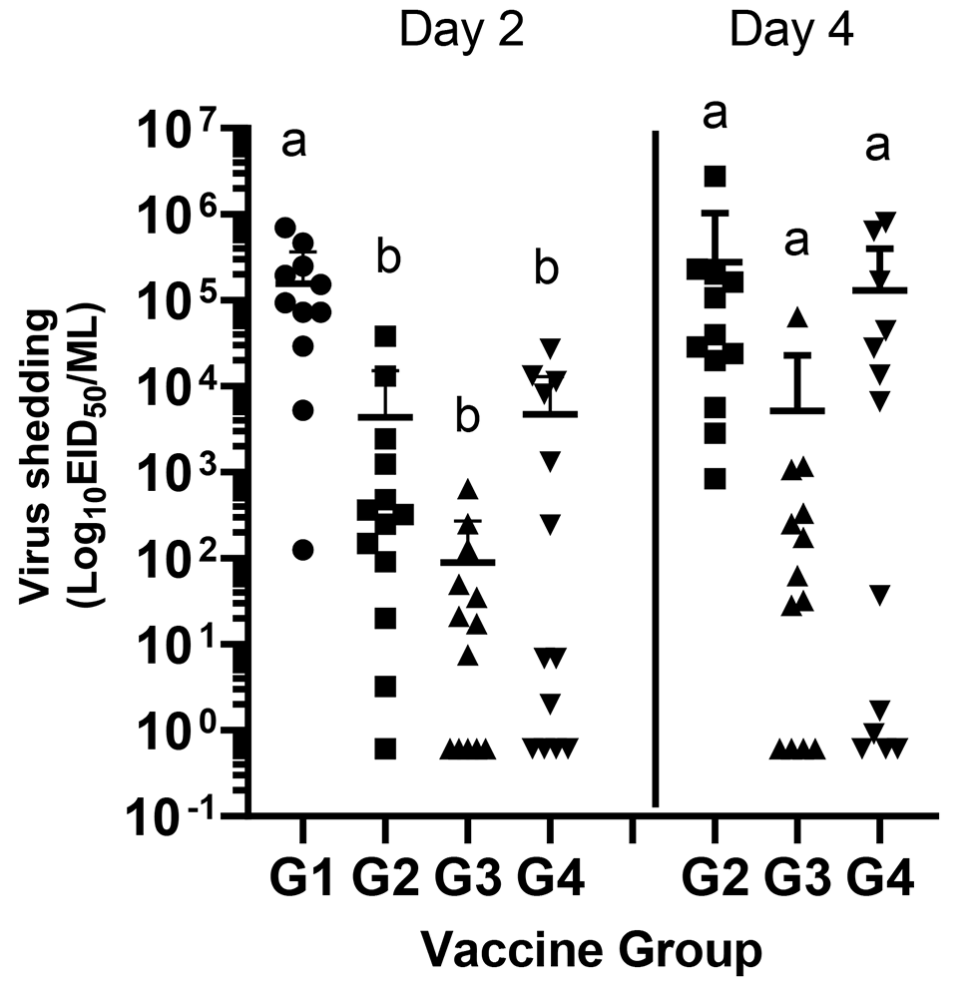
Viral titers from oral swabs in broiler chicks immunized at one-day of age with recombinant vaccine and challenged with HPAI virus in experiment 2. One-day old commercial chicks were vaccinated SQ with PBS (G1), RP-H5 (10^7^) (G2), RP-H5 (10^8^) (G3), or RG-H5 (G4). Birds were challenged with A/turkey/Minnesota/12,582/2015(H5N2) clade 2.3.4.4 HPAI virus at 3 weeks of age. Oral swabs were taken at 2 and 4 days post-challenge. Viral titers are expressed as log_10_ EID_50_/ml. Statistical differences are indicated by different lowercase lettering (*P* < 0.05)

Quantitation of viral shed was performed by qRRT-PCR using extrapolation of a standard curve generated with the challenge virus. Oral and cloacal swab samples from challenged birds were collected on 2 and 4 dpc. Virus shedding for experiment 1 and experiment 2 is shown in Figs. 3 and 4, respectively. In experiment 1, the rHVT-AI administered *in ovo* (G3) significantly reduced shedding compared to sham (G1) and RP-H5 *in ovo* (G2) vaccinated birds at 2 dpc (Fig. 3A and B). Mean titers in sham and RP-H5 vaccinated birds were approximately 10^5.0–5.5^ EID_50_/ml at 2 dpc following challenge at 3 and 6 woa. In contrast, rHVT (G3) vaccinated birds demonstrated titers of approximately 10^1.1–1.8^ EID_50_/ml on 2 dpc in both trials. Groups of birds receiving combined RP-H5 and rHVT-AI at one day of age (G5) also demonstrated significantly reduced shedding titers (between 10^1.2 and 2.0^ EID_50_/ml) compared to sham (G1) and RP-H5 (G2) *in ovo* vaccinated birds on 2 dpc in both trials. No differences were observed between the *in ovo* rHVT-AI (G3) and day of age vaccinated birds (G5) at either challenge time, or either day tested. Finally, groups of birds *in ovo* vaccinated and boosted with the RP-H5 vaccine (G4) demonstrated significantly reduced shedding titers of approximately 10^1.5^ EID_50_/ml compared to *in ovo* vaccinated sham and RP-H5 groups when challenged at 6 woa. At 4 dpc, no differences in shedding levels were observed in survivors of any group, regardless if challenged at 3 or 6 woa (Fig. 3A and B).

In experiment 2, significantly, higher virus shedding was observed between sham (G1) and all vaccinated groups (G2 (RP-H5 10^7^), G3 (RP-H5 10^8^), G4 (RG-H5)) at 2 dpc (Fig. 4). Titers in sham-vaccinated group contained a mean average level of 10^5.2^ EID_50_/ml. In contrast, the mean shedding titers from G2, G3, and G4 contained 10^3.6^, 10^1.9^ and 10^3.7^ EID_50_/ml, respectively. At day 4 pc, no significant difference in titers was observed in titers between the groups, with mean levels varying between 10^3.7^ and 10^5.4^ EID_50_/ml. It should be noted that most of the mortality observed in all vaccinated groups in this experiment occurred after day 4 pc (14/17).

### Serum hemagglutination titer (HI) in broiler chicks immunized and challenged with H5N2 HPAIV in experiment 1 and 2

**Fig. 5:**
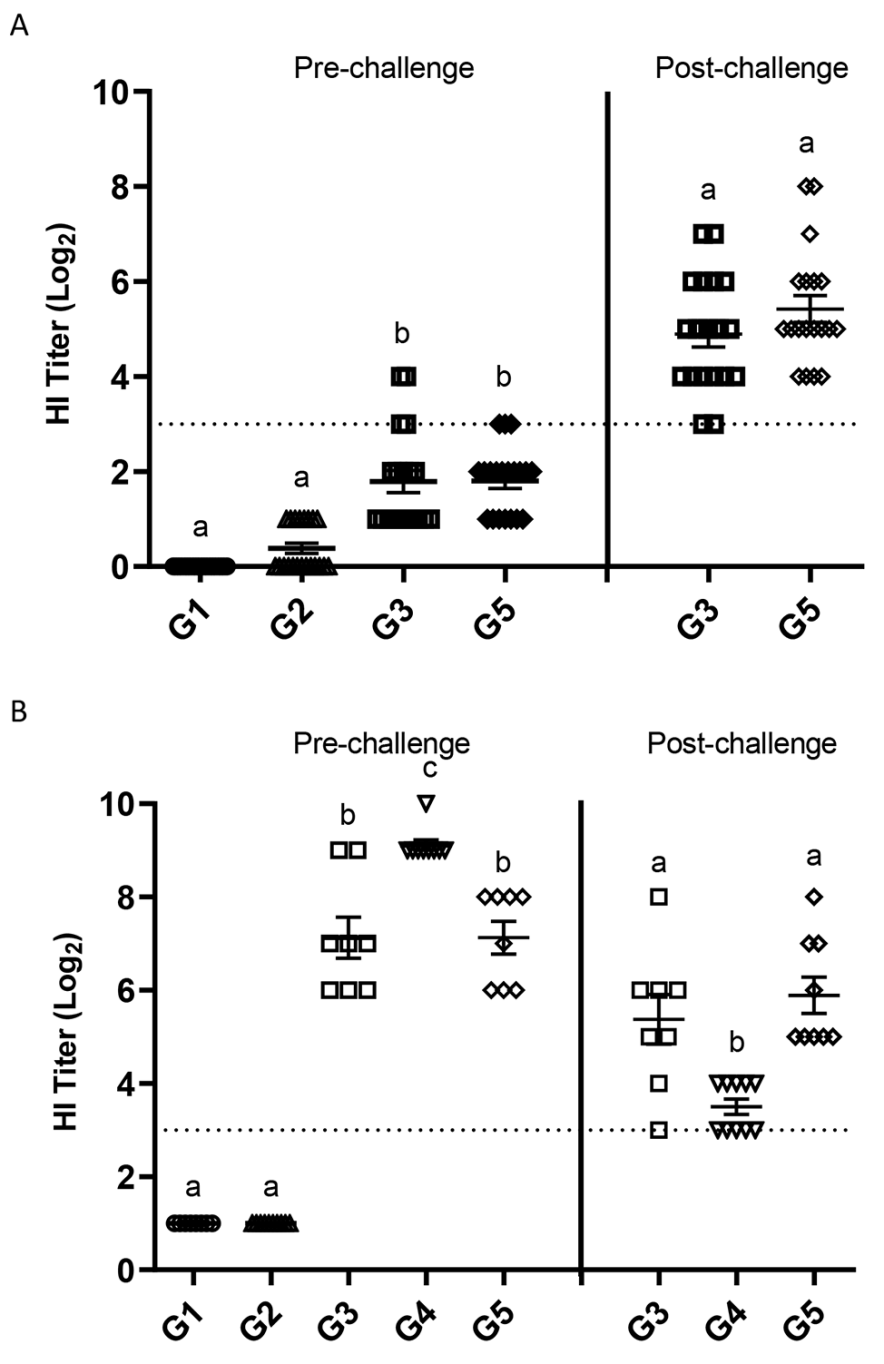
Serum HI titers (log_2_) in broiler chicks immunized with recombinant vaccine and challenged with HPAI in experiment 1. G1, G2, G3: 18 d old embryonated eggs were vaccinated *in ovo* with PBS (Sham control; G1) or RP-H5 (G2) or rHVT-AI (G3). G4: Birds in G2 group were given an additional RP-H5 booster dose at 3 weeks of age (G4). G5: Day old birds were vaccinated with both rHVT-AI and RP-H5 vaccine. Birds were challenged with HPAI virus at either 3 weeks of age (Fig. 5**A**) or 6 weeks of age (Fig. 5**B**). Serum samples were taken on day 0 (Pre) and day 14 (Post) after challenge. Statistical differences are indicated by different lowercase lettering (*P* < 0.05)

Serum samples were obtained for determination of antibody titer against the challenge virus both pre-and post-challenge. In experiment 1, in groups of birds challenged at 3 woa, no detectable positive titers (≥ 3 log_2_) were observed in sham (G1) or RP-H5 (G2) *in ovo* vaccinated birds (Fig. 5A). A few birds in the rHVT-AI (G3) and RP-H5/rHVT-AI combined (G5) vaccinated groups had positive HI titers pre challenge. All surviving birds in these groups seroconverted with positive HI titers of approximately 5 log_2_ on 14 dpc (Fig. 5A). In groups of birds challenged at 6 woa, no detectable positive titers were observed in sham (G1) or RP-H5 (G2) *in ovo* vaccinated birds (Fig. 5B). Birds *in ovo* vaccinated with rHVT-AI (G3) had mean HI titers of 7 log_2_ pre-challenge that dropped to 5.5 log_2_ at 14 dpc. Groups of birds that received RP-H5 *in ovo* and were boosted at 3 woa (G4) had mean HI titers of 9 log_2_ pre-challenge that decreased to 4 log_2_ post-challenge. Finally, groups of birds that received the combined RP-H5 and rHVT-AI (G5) demonstrated mean HI titers of approximately 7 log_2_ pre-challenge that decreased to 6 log_2_ by 14 dpc (Fig. 5B).

**Fig. 6:**
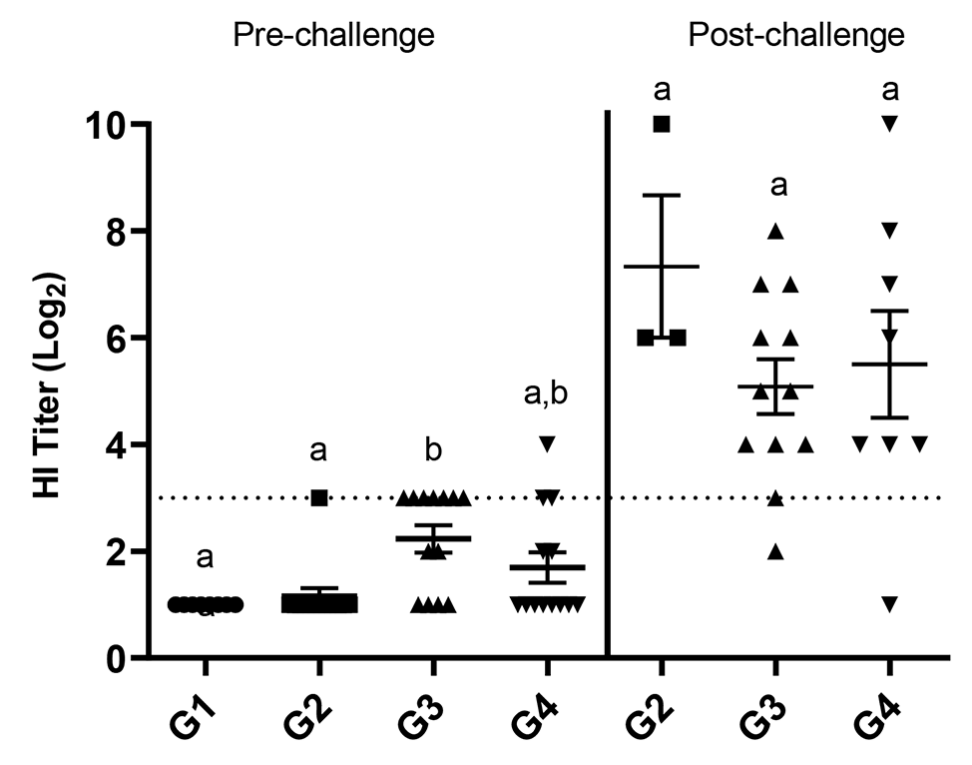
Serum HI titers (log_2_) in broiler chicks immunized with recombinant vaccine and challenged with HPAI in experiment 2. One-day old commercial chicks were vaccinated SQ with PBS (G1), RP-H5 (10^7^) (G2), RP-H5 (10^8^) (G3), or RG-H5 (G4). Birds were challenged with A/turkey/Minnesota/12,582/2015(H5N2) clade 2.3.4.4 HPAI virus at 3 weeks of age. Serum samples were taken on day 0 (Pre) and day 10 (Post) after challenge. Statistical differences are indicated by different lowercase lettering (*P* < 0.05)

In experiment 2, pre-challenge HI titers were significantly higher in RP-H5 (10^8^) (G3-2.2 log_2_) and RG-H5 (G4-1.7 log_2_) compared to sham (G1-1.0 log_2_) and RP-H5 (10^7^) (G2-1.2 log_2_) (Fig. 6). Few birds in any group demonstrated positive HI titers (≥ 3 log_2_) prior to challenge, with G3 containing the most (7/13). The increased pre-challenge HI titers in this group correlated with highest survival. After challenge, the HI titers increased in all vaccinated groups. In G2, the highest mean HI titers were observed (7.3 log_2_) which correlated with the highest virus shedding (Fig. 4) and highest mortality after challenge (Fig. 2). For comparison, titers in G3 and G4 after challenge were 5.1 log_2_ and 5.5 log_2_, respectively (Fig. 6).

## Discussion

Vaccination of poultry is a cost-effective approach to disease prevention; however, its application for AI is not yet globally warranted for a myriad of reasons [19, 20]. Besides cost, trade issues and lack of protective immunity to prevent infection and transmission are key considerations against AI vaccination. In addition, the overwhelming majority of vaccines, including inactivated and recombinant, provide systemic immunity, but not mucosal immunity, and thus do not prevent infection [12]. The proper use of vaccines, as a component of an AI control strategy, must be added to traditional control methods (e.g., stamping out, animal movement control, increased biosecurity, and increased surveillance). AI vaccines should be antigenically matched to the field virus, and ideally would be amenable to mass-administration techniques to large numbers of poultry to make them more cost effective [21].

*In ovo* vaccination is an efficacious and convenient method of vaccinating embryonating embryos in the hatchery with over 80% of US broiler industry utilizing this approach to control Marek’s disease virus [22]. Advantages of *in ovo* vaccine include decreased labor, time, and costs and facilitates uniform administration of vaccine dose into hundreds of eggs per minute. A consideration for this type of vaccination program is the shorter life span of broiler type birds. Earlier studies have successfully demonstrated efficacy following *in ovo* vaccination with AI challenge [23, 24]. In addition, numerous experimental studies have demonstrated the efficacy of the rHVT-AI vaccine in poultry, with protection from HPAI ranging from 60 to 100% [7, 25–30]. The rHVT-AI vaccine appears to induce lower antibody titers compared to traditional inactivated vaccines [25]. However, in these studies the antigen used for challenge was not matched to the rHVT-AI insert used for vaccination, or the HI test, and therefore this may have contributed to lower titers observed in these studies and others [7, 8, 25, 27]. We have previously demonstrated these types of recombinant vaccines induce cross-reactive cellular immunity to the HA, with broader protection from various H5 HPAI lineages [25]. The combined humoral and cellular immunity in that study resulted in significantly reduced viral shedding after challenge.

The aim of this study was to investigate the protective efficacy of three recombinant H5 vaccines for application in commercial broilers against North American clade 2.3.4.4c H5N2 HPAI virus challenge. Overall, the results demonstrate that *in ovo* vaccination with a single dose of the rHVT-AI or two doses of RP-H5 adequately protected the birds and decreased the virus shedding. The results also demonstrate that the RP-H5 did not induce immunity when delivered only *in ovo*, based on serology results and lack of protection, and may indicate the vaccine is not suited for *in ovo* application. The RP-H5 was not designed for use in ovo application, but we included it as an option because to see if it could facilitate a mass vaccination approach if this route of administration was effective. It is worth noting that antigenic matching did not appear to offer increased protection as both the RG-H5 and RP-H5 given alone did not demonstrate better protection. Finally, combining both vaccines (rHVT-AI and RP-H5) and applying them at one day of age also provided good protection. It should be noted that a higher challenge dose of virus was used based on a higher bird infectious dose of North American 2.3.4.4c H5 viruses determined by Bertran, et al. [5]. This higher dose may have contributed to higher levels of virus shedding observed in vaccinated groups after challenge.

The recombinant alphavirus vaccine, RP-H5, has also been demonstrated to provide protection against HPAI challenge, although better protection has been demonstrated when this vaccine is used in a prime-boost schedule [8, 9, 31, 32]. The results here demonstrated the inability of the vaccine to provide protection when delivered *in ovo*. These results occurred most likely from a failure of antigen expression and/or induced immunity in immunologically immature animals, as no detectable antibodies were observed pre-challenge. One possibility was the vaccine was delivered to the amniotic fluid rather parenterally into the bird. Delivering the vaccine inoculum to the amniotic fluid is technically an oral vaccine and this route of delivery could have contributed to the failure of protection. Providing a second dose of RP-H5 vaccine subcutaneously at 3 weeks of age decreased viral shedding, increased antibody levels, and protected against HPAI challenge at 6 weeks of age. When the vaccines were applied (rHVT-AI and RP-H5) in combination at one day of age, protection at 6 weeks of age with decreased virus shedding was observed. However, it is difficult to assess the contribution of the RP-H5 in this schedule as *in ovo* application with the rHVT-AI alone provided protection at 6 weeks.

Reverse genetics derived vaccines for avian influenza have been used globally for many years in countries that allow for AI vaccination, including China, Egypt, Hong Kong, Indonesia Vietnam, and Mexico [33]. One advantage to these vaccines is the ability to directly match the HA in the vaccine to the outbreak strain. In our previous studies, the RG-H5 vaccine provided complete protection against HPAI challenge in chickens and turkeys when vaccinated at 3 weeks of age [7, 8]. In these studies, vaccination at one-day of age only provided some protection (62%) against challenge. It is likely that age of the bird at vaccination, not being immunologically mature, contributed to the lack of protection observed in these studies compared to the previous studies because the birds in all three studies received an equal dose and adjuvant for vaccination as well as the challenge isolate and dose. Previous vaccination studies of day old chicks support the idea that their immune response is greatly reduced and the immune response will be reduced [34, 35]. The RP-H5 vaccine did demonstrate a dose-based difference in immune response with birds receiving the 10^8^ vaccine dose had better protection than the 10^7^ dose, but it still wasn’t completely protective. Despite the obvious advantages of vaccinating birds in the hatchery, the use of killed or non-replicating vaccines has severe limitations when working with day old chicks.

In conclusion, this study provides support for the use of recombinant vaccines for protection of broiler chickens should a similar outbreak occur, and emergency vaccination is considered. During the recent U.S. outbreaks of clade 2.3.4.4 HPAI, vaccination was considered, however many variables such as trade issues, lack of vaccine efficacy studies, and commodity group interest, resulted in non-deployment in the field. While not tested in these studies, use of a DIVA strategy to distinguish vaccinated from infected animals can be utilized with recombinant vaccine technology [36–38]. The ability to utilize active surveillance in vaccinated flocks is a critical consideration of a vaccination program. Although nearly 47 million birds died or were destroyed in recent outbreaks, the triggers to employ AI vaccination in the U.S are not defined. Here, vaccination with the rHVT-H5 *in ovo* provided the best protection. While two doses of RP-H5 provided protection, the goal of a broiler vaccination program would be to achieve protection in a single application. These data provided support for consideration of recombinant vaccine protection of broiler birds from the current H5Nx HPAI outbreak.

## Conclusion

Highly pathogenic avian influenza (HPAI) virus is a constant threat to the poultry industry. Between December 2014 and June 2015, the U.S. experienced a HPAI outbreak with approximately 47 million birds dead or euthanized from infection with a H5Nx clade 2.3.4.4c virus. The economic impact of this outbreak is an estimated $3.3 billion USD to the U.S. poultry industry and resulted in trade partners banning U.S. poultry imports. During the outbreak, the ability of vaccines to protect commercial chickens from HPAI was unknown. In this study, we investigated three recombinant H5 avian influenza vaccines for protection of broiler chickens. The results demonstrate that while all were effective, one was superior when delivered *in ovo* into the developing embryo. This route of vaccination would be considered optimal since it can be applied in a mass application to commercial poultry. Overall, these data can be used to develop intervention strategies to protect commercial broiler flocks from future HPAI outbreaks.

## Data Availability

All data generated or analyzed during this study are included in this published article.

## Abbreviations

ABSL3E: Animal biosafety level 3 enhanced
AI: Avian influenza
AIV: Avian influenza virus
DIVA: Differentiating infected versus vaccinated animals
DPC: Days post–challenge
EID_50_: 50% embryo infectious dose
HA: Hemagglutinin
HAU: Hemagglutinin Units
HI: Hemagglutinin inhibition
HPAI: Highly pathogenic avian influenza
IACUC: Institutional Animal Care and Use Committee
IN: Intranasal
LPAI: Low pathogenic avian influenza
PDRC: Poultry Disease Research Center
qRRT: PCR–Quantitative real time RT–PCR
RG: H5–Reverse genetics derived vaccine expressing H5 hemagglutinin gene
rHVT: AI–Recombinant herpesvirus of turkey encoding H5 hemagglutinin gene
RP: H5–Replication–deficient alphavirus RNA particle vaccine encoding H5 hemagglutinin gene
SEM: Standard Error of the Mean
SQ: Subcutaneous
USDA: United States Department of Agriculture
USNPRC: United States National Poultry Research Center
WOA: Weeks of age
